# Accuracy and prognostic value of radiological lymph node features in variant histologies of bladder cancer

**DOI:** 10.1007/s00345-022-04010-6

**Published:** 2022-04-23

**Authors:** Severin Rodler, Olga Solyanik, Maria Ingenerf, Matthias Fabritius, Gerald B. Schulz, Friedrich Jokisch, Yannic Volz, Thilo Westhofen, Benedikt Ebner, Jozefina Casuscelli, Alexander Kretschmer, Raphaela Waidelich, Boris Schlenker, Christian Stief, Alexander Buchner, Lennert Eismann

**Affiliations:** 1 Department of Urology, Ludwig-Maximilians-University, Marchioninistr.15, 81377 Munich, Germany; 2 Department of Radiology, Ludwig-Maximilians-University, Munich, Germany

**Keywords:** Bladder cancer, Variant histology, Computed tomography, Lymph node staging

## Abstract

**Purpose:**

To provide first evidence of lymph node (LN) staging using CT scan and its prognostic value in variant histologies of bladder cancer. This knowledge may optimize patient management with variant histologies based on CT morphological findings.

**Methods:**

Preoperative CT scans of patients with variant histologies who underwent RC between 2004 and 2019 were reanalyzed by two independent radiologists in a blinded review process. Specificity, sensitivity, and accuracy for LN staging as well as LN characteristics were evaluated. Correlation with survival was investigated by Kaplan–Meier method, log-rank test and multivariate analysis.

**Results:**

1361 patients with primary tumor of the bladder underwent RC, of which 163 (12%) patients revealed variant histologies. 65 (47.8%) patients have shown an urothelial variant (UV) and 71 (52.2%) a non-urothelial variant (NUV). LN metastases were found in 18 (27.7%) patients with UV and 21 (29.6%) patients with NUV. The accuracy to detect LN metastasis for all variant histologies was 62% with a sensitivity of 46% and a specificity of 70%. Subgroups of UV and NUV revealed an accuracy of 67% and 57%. An increased number of regional LN (HR 2.8; 1.34–6.18) and the loss of fatty hilum (HR 0.36, 0.17–0.76) were prognostic parameters. In multivariate analysis, a fatty hilum (HR 0.313, 0.104–0.945) and the presence of lymph node metastases (HR 2.866, 1.140–7.207) were prognostic.

**Conclusion:**

This first study on CT morphological behavior of variant histologies revealed an accuracy of UV and NUV comparable to UC with low specificity for all variant histologies. CT scan prior RC should be interpreted in regard to histological subtypes.

**Supplementary Information:**

The online version contains supplementary material available at 10.1007/s00345-022-04010-6.

## Introduction

Bladder cancer accounts for 4.5% of all newly diagnosed cancers in the US and is more prevalent in men than women [1]. 75% of all cases present as non-muscle invasive bladder cancer (NMIBC) and are treated locally by transurethral resection of the bladder (TUR-B) followed by adjuvant intravesical instillation therapy [2]. As soon as the disease presents as muscle invasive bladder cancer (MIBC) or high risk NMIBC, the treatment of choice is radical cystectomy (RC) with a median 5-year-overall survival of 50% [3]. However, for metastatic urothelial carcinoma (UC), systemic treatment is recommended with a median overall survival dropping to 16 months even in the latest trials [4].

Variant histologies account for up to 25% of all bladder cancers [5]. They can be subcategorized into urothelial and non-urothelial variants depending on the detection of urothelial carcinoma cells in the histology [6]. Urothelial variants included urothelial carcinoma with squamous differentiation (UCSD), sarcomatoid (SARCO), micropapillary and plasmacytoid variant. Non-urothelial and pure histological variants include squamous cell carcinoma (SCC), glandular variant, neuroendocrine carcinoma, and adenocarcinoma (AC) [5]. Although survival of most variant histologies is not statistically different from pure UC [7], neuroendocrine, signet ring cell and spindle cell tumors reveal a poor prognosis [8]. However, variant histologies are often diagnosed at more advanced stages than UC and cohorts published in the literature are often small [9].

Preoperative staging is essential to advise patients for the most effective treatment option. Nodal metastases as a high-risk feature might guide neoadjuvant chemotherapy in chemosensitive urothelial variants of bladder cancer. Patients with high risk of tumor progression might also be triaged to neoadjuvant or inductive immunotherapy as an alternative treatment regime [10]. In pure UC, contrast-enhanced computed tomography (CT) scan is the gold standard staging modality prior to RC. However, sensitivity of CT scan for detection of lymph node metastasis is not optimal (48–87%) [11]. Although representing 25% of all cases, accuracy of preoperative imaging regarding detection of lymph node metastasis and prognostic features of LN in variant histologies are completely unclear. Results from UC might not be directly transferable as the biological behavior and metastatic spread are found to differ between variant histologies [12]. Patients with variant histologies tend to present with advanced disease [5], and a precise staging and assessment of LNs seems paramount. To date, there is no evidence at all for staging using CT scan in variant histologies of the bladder prior RC.

Therefore, we aim to investigate the impact of positive LN at the time of surgery on survival, the accuracy, sensitivity and specificity of lymph node detection in preoperative contrast enhanced CT imaging and prognostic features of LNs in variant histologies of bladder cancer prior RC.

## Materials and methods

### Setting

We performed a retrospective analysis of all patients who underwent RC at our academic center between 2004 and 2019. Indication for RC were MIBC, BCG-refractory NMIBC after exclusion of distant disease (cM0) or palliative reasons, according to the guidelines of the EAU [11]. RC was performed by trained urologists according to a standardized surgical procedure including pelvic lymphadenectomy and urinary diversion by ileal neobladder or ileal conduit. All patients received standard regional lymph node dissection according to EAU guidelines with a cranio-caudal extent from the common iliac bifurcation to the circumflex iliac vein and a medio-lateral extent from the ureter to the genitofemoral nerves [3]. Histological specimen was worked up by our experts of the *Department of Pathology for urogenital malignancies*. Classification was performed according to the latest TNM Classification of Malignant Tumors, UICC-classification and WHO classification [6, 13]. Post-operative rehabilitation and psycho-oncological care was offered to all patients.

### Preoperative imaging

All patients received a contrast-enhanced abdominal and pelvic CT scan prior to RC. Images were acquired within 6 weeks prior RC. In case neoadjuvant chemotherapy was given, the last imaging prior RC was used for further analysis. The slice thickness used to reconstruct images for retrospective review was at least 5.0 mm. Image analysis for this study was conducted in single-blinded review process performed by two radiologists specialized in genitourinary imaging. The two radiologists reanalyzing the images had neither access to the pathological report nor the initial radiological report. After completion of all cases, results were compared by our research group. In cases of discrepancy consensus was conducted for final decision. Review of images was performed on Picture Archiving and Communication System (PACS). To interpret the metastatic involvement of lymph nodes the following parameters on CT scan were evaluated: a size of more than 8 mm in the short axis, the loss of fatty hilum and the normal reniform lymph node shape with a more rounded or irregular configuration. Furthermore, we reported increased number of regional lymph nodes (NLN) irrespectiv of lymph node´s shape and size as an additional characteristic.

### Follow-up

Follow-up was based on the recommendation of the EAU guidelines and carried out either by office-based urologists or our specialized outpatient clinic [11]. Therefore, we performed a structured follow-up by questionnaires regarding quality of life, impact of urinary diversion and report of latest imaging to update oncologic status. Follow-up letters were sent twice in the first year after RC and then annually. Written informed consent to be included into this study and to provide follow-up data was obtained from all patients following the World Medical Association Declaration of Helsinki [14]. The institutional ethic committee of Ludwig-Maximilians-University of Munich approved this study prior initiation (Reference number 20-179).

### Statistics

Kaplan–Meier analysis, logrank test and univariate as well as multivariate Cox regression models were used for outcome analysis with the endpoints cancer specific survival (CSS) and overall survival (OS). Continuous variables were compared between groups using Kruskal–Wallis test. For comparison of categorial parameters the chi-square test was used. Sensitivity, specificity and accuracy were calculated for detection of LN metastasis. Receiver operating characteristics (ROC)-analysis was used to calculate optimal size cut-offs. *p* values smaller than 0.05 were regarded as significant. All calculations were performed using the software MedCalc version 20 (MedCalc Ltd., Ostend, Belgium).

## Results

### Patient characteristics

From 2004 to 2020, 1672 patients underwent radical cystectomy at our institute and 1361 due to bladder cancer. 1198 (88%) patients revealed pure urothelial carcinoma and 163 (12%) variant histology of bladder cancer in final specimen. We report 59 patients with squamous carcinoma (43.4%), 55 patients with urothelial squamous dedifferentiated carcinoma (40.4%), 12 patients with adenocarcinoma (8.8%) and 10 patients with sarcomatoid carcinoma (7.4%) of the bladder. 27 (16.6%) patients revealed other variant histologies (Supplementary Fig. 1).

Overall follow-up of the study cohort was 21 months (IQR 9–48). 2-year OS and 2-year CSS is reported with 55% and 57% for all variant histologies. Distinguishing urothelial and non-urothelial variants 2-year CSS were 58% and 57%. Subgroups showed the following 2-year OS; CSS: 51%; 53% in SCC, 57%; 58% in UCSD, 62%; 73% in AC and 56%; 56% in SARCO (Supplementary Fig. 2 and Table 1).

**Table 1:** Patient characteristics

	Urothelial variants				Non-urothelial variants				*p*-value
	UCSD		SARCO		SCC		AC		
	%	*n*	%	*n*	%	*n*	%	*n*	
Age									0.777
Median	69		60		64		71		
IQR	57–76		54–76		57–76		64–75		
Follow-up									0.463
Median	23		16		20		27		
IQR	10–48		4–32		5–83		21–40		
Year of presentation									0.002
Median	2013		2016		2011		2015		
IQR	2009–2016		2014–2017		2008–2017		2010–2017		
Gender									0.184
Male	62	34	40	4	47.5	28	33	4	
Female	38	21	60	6	52.5	31	67	8	
ASA score									0.535
1	0	0	0	0	5	3	0	0	
2	42	23	30	3	32	19	33	4	
≥ 3	58	32	70	7	63	37	67	8	
Histology TUR-B									0.208
Equal to RC	89	49	100	10	97	57	100	12	
Unequal to RC	11	6	0	0	3	2	0	0	
Neoadjuvant chemotherapy									0.576
Yes	2	1	0	0	5	3	8	1	
No	98	54	100	10	95	56	92	11	
Adjuvant chemotherapy									< 0.001
Yes	0	0	20	2	5	3	33	4	
No	100	55	80	8	95	56	66	8	
*T*									0.255
≤ pT2	25.5	14	20	2	19	11	0	0	
pT3–4	74.5	41	80	8	81	47	100	12	
*N*									0.573
pN0	60	33	60	6	61	36	50	6	
pN+	29	16	20	2	25	15	50	6	
pNX	11	6	20	2	14	8	0	0	
*M*									0.076
*M*0	87	48	90	9	93	55	67	8	
*M*1	13	7	10	1	7	4	33	4	
*R*									0.419
*R*0	87	48	90	9	76	45	83	10	
*R*1	13	7	10	1	24	14	17	2	
Number of LN removed									0.296
0	11	6	20	2	14	8	0	0	
1–10	31	17	30	3	20	12	58	7	
11–20	44	24	40	4	46	27	17	2	
21–30	15	8	10	1	20	12	25	3	
Number of LN positive									0.714
0	67	33	75	6	71	36	50	6	
1–10	33	16	25	2	27	14	50	6	
11–20	0	0	0	0	2	1	0	0	
21–30	0	0	0	0	0	0	0	0	
Urinary diversion									0.484
Ileum conduit	58	32	80	8	75	44	75	9	
Ileum neobladder	40	22	20	2	25	15	25	3	
Pouch	2	1	0	0	0	0	0	0	

*UCSD* urothelial carcinoma with squamous differentiation, *SARCO* sarcomatoid urothelial carcinoma, *SCC* squamous cell carcinoma, *AC* adenocarcinoma, *IQR* interquartile range, *n* number of patients, *p* *p*-value, *ASA* American Society of anaesthesiology Physical Status Classification System, *TUR-B* transurethral resection of bladder

### Prevalence of lymph node metastases in variant histologies and their impact on survival

**Fig. 1:**
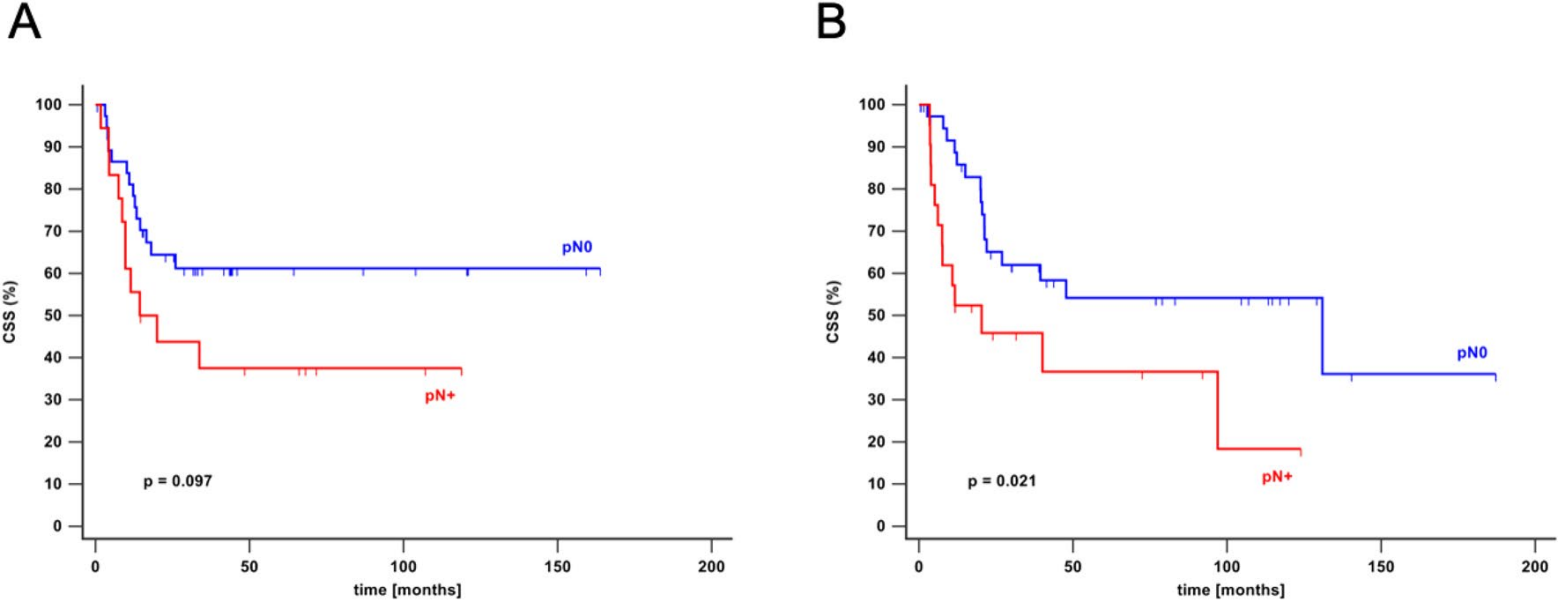
Cancer specific survival for urothelial and non-urothelial variants of bladder cancer depending on lymph node status. Cancer specific survival (CSS) for urothelial (**A**) and non-urothelial (**B**) variants of bladder cancer stratified by detection of lymph node metastases (pN+) and lymph node negative patients (pN0). Statistical analysis was performed by Kaplan–Meier method. *CSS* cancer specific survival, *pN+* lymph node metastasis, *pN0* no lymph node metastasis

Lymph node metastasis was found in 15 (25.4%) patients with SCC, 16 (29.1%) patients with UCSD, 6 (50.0%) patients with AC and 2 (20.0%) patients with SARCO (Table 1). 2-year CSS for patients with urothelial variants of bladder cancer was 64% for patients with negative lymph nodes (N0) and 44% for patients with positive lymph nodes (N1, N2). For non-urothelial variants 2-year CSS was 65% for patients without lymph node involvement (N0) and 46% for patients with nodal metastasis (N1, N2) (Fig. 1).

### Diagnostic value and accuracy of CT scan in variant histologies of bladder cancer

Accuracy to detect LN metastasis in preoperative CT scan for variant histologies was 62% with a sensitivity of 46% and a specificity of 70%. Subgroups of urothelial variants and non-urothelial variants revealed an accuracy of 67% and 57%, a sensitivity of 53% and 41%, and specificity of 73% and 68% (Table 2).

**Table 2:** Accuracy of preoperative CT scans

	Accuracy		Sensitivity		Specificity	
	%	*n*	%	*n*	%	*n*
Variant histologies	62	67/108	45	17/38	71	50/70
Urothelial variants	67	35/52	44	8/18	79	27/34
Non-urothelial variants	57	32/56	45	9/20	64	23/36
SCC	58	26/46	50	7/14	61	19/31
AC	55	6/11	33	2/6	80	4/5
UCSD	68	30/44	50	8/16	79	22/28
SARCO	63	5/8	0	0/2	83	5/6

*UCSD* urothelial carcinoma with squamous differentiation, *SARCO* sarcomatoid urothelial carcinoma, *SCC* squamous cell carcinoma, *AC* adenocarcinoma

### Prognostic value of CT scan in variant histologies of bladder cancer

**Fig. 2:**
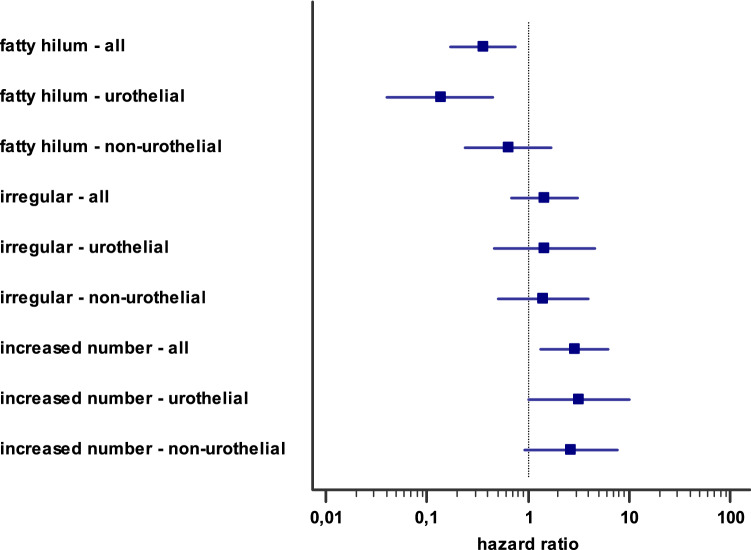
Prognostic value of CT morphological characteristics on survival. Hazard ratio of single CT morphological characteristics of lymph nodes in regard of cancer specific survival

Morphological characteristics of lymph nodes were evaluated in size, shape, fatty hilum and regional amount. Across the whole study cohort, we observed an increased number of loco-regional lymph nodes (HR 2.8; 1.34–6.18) and the loss of fatty hilum (HR 0.36, 0.17–0.76) as prognostic parameters with statistical significance (Fig. 2, Supplementary Fig. 3). In multivariate analysis, fatty hilum (HR 0.313, 0.104–0.945) and lymph node metastases (HR 2.866, 1.140–7.207) were significantly correlated with survival (supplementary table 1). The median maximum size of all lymph nodes detected was 9 mm (IQR 6–12 mm). 8 mm was found to be an optimal cut-off [area under the curve (AUC): 0.713)] with as sensitivity of 82%, a specificity of 59% and an accuracy of 67% (Supplementary Fig. 4).

## Discussion

This is the first study to investigate the value of preoperative CT staging in regard of accuracy, prognostic value and differences among the individual variant histologies of bladder cancer. Histological confirmed LN metastases are associated with poor outcome. However, accuracy for LN metastases in variant histologies is 62% with a higher specificity than sensitivity. Fatty hilum and increased number of loco-regional LNs were associated with worse oncological outcomes.

### Different tumor biologies and routes of metastases

Variant histologies account for up to 25% of all malignancies of the bladder but evidence of tumor biology is still limited as studies are highly heterogenous [15]. Due to major differences in tumor biology of histologic variants the lymphatic metastatic spread was reported to differ among histological origin [12]. Additionally, histologic variants are associated to local inflammation and might lead to reactively enlarged LN [15]. Consequently, reactive LN might be misinterpreted in CT scan as a metastatic disease stage, thus making diagnostic work-up more challenging than in pure urothelial carcinoma patients [16]. We observed LN metastases rates between 20% in SARCO and 50% in AC of the bladder.

### Survival of patients with lymph node metastases in variant histologies of bladder cancer

Poor survival of patients with LN involvement is observed in variant histologies of bladder cancer similar to pure urothelial carcinoma. Standard of care is still pathological tumor staging of LNs simultaneously performed by RC. Extend of lymphadenectomy is a controversial topic but Herr et al*.* introduced the ratio of positive LNs to all removed LNs as a quality parameter of lymphadenectomy [17]. We performed in our cohort standard regional LN dissection as described [3]. 5-year CSS in patients with variant histologies drops from 82.5 to 41.9% when LN metastasis are detected [18]. Perioperative chemotherapy is standard of care for pure urothelial carcinoma, whereas survival benefits for histological variants remains uncertain [19]. Therapy management should be adjusted to histological subclassification [20]. Patients with clinical suspicious LNs are recommended to be treated by systemic therapy and might undergo RC for consolidation [21]. Even though pure UC has been well investigated, 5-year survival rates of cN+ patients shows a wide range from 10 to 59% [21]. Accordingly, cN+ patients revealing variant histologies present a patient clientele with uncertain risk of progression. Therefore, our data present complementary survival rates of a high-volume center and demonstrate the high unmet medical need to improve risk stratification of cN+ variant histologic bladder cancer.

### Accuracy, specificity and sensitivity of CT scans in bladder cancer

CT scan is the gold standard for clinical staging of bladder cancer regardless of the presence of variant histologies [3]. Clinical staging is crucial to evaluate LN status or detect distant metastases. Even though CT scan is very limited in identifying tumorous LN and is reported with an accuracy ranging from 61 to 86%, a higher specificity (93.6%) than sensitivity (52.6%) was observed [22]. However, the lack of detecting LN metastases might be most challenging in normal sized LNs [22, 23]. The current research has exclusively focused on pure urothelial carcinoma or not differentiated in cases of variant histologies [22]. To the current knowledge there is no evidence of accuracy on urothelial or non-urothelial variants despite its essential impact on clinical decision making. In our study, the presented accuracy of 67% in urothelial and 57% in non-urothelial variants are comparable to staging CT scan in pure urothelial carcinoma. Interestingly, a low sensitivity below 50% is observed in the overall study cohort, revealing a high unmet medical need for better preoperative staging as well as potential inclusion of alternative imaging modalities for patients with variant histologies. We found 8 mm to be an optimal cut-off for accuracy of lymph node metastasis detection in variant histologies in our ROC analysis. Thereby, we are the first to report this cut-off in variant histologies that is recommended by guidelines for pure UC [11].

### Prognostic lymph node characteristics

In view of the fact, that even presence of LN metastasis shows a wide variation in survival, we evaluated the prognostic value of CT morphologic criteria to better risk stratify patients prior their surgical therapy. For classical urothelial carcinoma different morphological features in preoperative imaging have been investigated to predict lymph node metastases and to predict survival [24]. To date there is no evidence for such features in variant histologies. Perioperative treatment options for patients with variant histologies are scarce but due to higher tumor stages at time of initial diagnosis and poor prognosis its impact is substantial for survival [15]. Therefore, the identification of high-risk patients in the preoperative staging is even more decisive to provide best patient care. For all variants the loss of fatty hilum was associated with decreased CSS and OS and subgroup analysis revealed a significant impact for urothelial variants only. Likewise, the increased number of locoregional LN was related to poor survival rates.

### Clinical consequence and future directions

According to our newly identified risk criteria in preoperative staging, patient might undergo risk-adapted therapy management. Chemosensitive variants presenting morphological features of high risk might benefit from neo-adjuvant chemotherapy before receiving radical surgery [8]. In contrast surgical treatment is the standard for SCC and early RC should be discussed to achieve best oncological results [25]. Consequently, preoperative staging of histological variants might be interpreted carefully in contrast to pure urothelial carcinoma. Morphological characteristics hold prognostic value in different histological variants and might help to identify patients of high risk for progression and limited lifetime. Further, alternative imaging modalities as FDG-PET CT with potentially higher sensitivity for detection of LN metastases might be applied for adequate staging in variant histologies of bladder cancer. Another interesting application might be enhancing the radiologist by modern technologies as neuronal networks and find further radiological features that predict lymph node metastases. Therefore, radiomics to extract novel features might be an ideal application [26].

### Limitations

In regard of the retrospective single-center character of this study the presented results hold limitations. Due to the status as an academic referral center the patient collective might differ from non-academic centers. Additionally, radiological evaluation was performed by two specialists of urogenital malignancies and was limited to the listed morphological criteria. Also, the case load especially for sarcomatoid variant is small and should be validated in larger multi-institutional trials. Thereby, researchers should focus on the impact of neoadjuvant chemotherapy on the performance of CT scan staging as our study is underpowered to address this question comprehensively. As the study has been performed retrospectively, lymph nodes resected and sent for pathology could not be matched with the precise localization of suspicious lymph nodes on CT imaging. Prospective trails are warranted to close this knowledge gap. However, we present one of the largest single-center cohorts with variant histologies. Most of the published cohorts of variant histologies are very limited in their number of patients or are created out of databases which do not hold primary clinical information such as full CT scans to enable analysis. Therefore, the presented study provides unique CT morphological information for variant histologies of the bladder.

## Conclusion

CT scan is the gold standard in preoperative staging prior RC. Its limitation of identifying LN metastases is well investigated for pure urothelial carcinoma but the presented results are first insights of CT morphological behavior of variant histologies. Accuracy of urothelial and non-urothelial variants are comparable to those reported for classic urothelial carcinomas, however, sensitivity is low. 8 mm is found to be the optimal cut-off value for detection of metastases. Loss of fatty hilum and increased number of loco-regional LNs hold prognostic potential in certain variants. Consequently, risk stratification among preoperative CT scan might improve management in patients with variant histologies and poor prognosis.

## Supplementary Information

Below is the link to the electronic supplementary material.

Supplementary file1 (DOCX 1331 kb)

## References

[1] Siegel RL, Miller KD, Jemal A (2020). Cancer statistics, 2020. CA Cancer J Clin.

[2] Babjuk M, Burger M, Compérat EM, Gontero P, Mostafid AH, Palou J (2019). European Association of Urology guidelines on non-muscle-invasive bladder cancer (TaT1 and carcinoma in situ)—2019 update. Eur Urol.

[3] Witjes JA, Bruins M, Compérat E, Cowan NC, Gakis G, Hernández V et al (2018) EAU guidelines on muscle-invasive and metastatic bladder cancer 2018. European Association of Urology Guidelines 2018 Edition. European Association of Urology Guidelines Office, Arnhem

[4] Galsky MD, Arija JÁA, Bamias A, Davis ID, De Santis M, Kikuchi E (2020). Atezolizumab with or without chemotherapy in metastatic urothelial cancer (IMvigor130): a multicentre, randomised, placebo-controlled phase 3 trial. Lancet.

[5] Moschini M, D'Andrea D, Korn S, Irmak Y, Soria F, Compérat E (2017). Characteristics and clinical significance of histological variants of bladder cancer. Nat Rev Urol.

[6] Humphrey PA, Moch H, Cubilla AL, Ulbright TM, Reuter VE (2016). The 2016 WHO classification of tumours of the urinary system and male genital organs-part B: prostate and bladder tumours. Eur Urol.

[7] Rodler S, Buchner A, Ledderose ST, Eismann L, Volz Y, Pfitzinger P (2020). Prognostic value of pretreatment inflammatory markers in variant histologies of the bladder: is inflammation linked to survival after radical cystectomy?. World J Urol.

[8] Veskimäe E, Espinos EL, Bruins HM, Yuan Y, Sylvester R, Kamat AM (2019). What is the prognostic and clinical importance of urothelial and nonurothelial histological variants of bladder cancer in predicting oncological outcomes in patients with muscle-invasive and metastatic bladder cancer? A European association of urology muscle invasive and metastatic bladder cancer guidelines panel systematic review. Eur Urol Oncol.

[9] Willis DL, Fernandez MI, Dickstein RJ, Parikh S, Shah JB, Pisters LL (2015). Clinical outcomes of cT1 micropapillary bladder cancer. J Urol.

[10] Necchi A, Raggi D, Gallina A, Madison R, Colecchia M, Lucianò R (2020). Updated results of PURE-01 with preliminary activity of neoadjuvant pembrolizumab in patients with muscle-invasive bladder carcinoma with variant histologies. Eur Urol.

[11] Witjes JA, Bruins HM, Cathomas R, Compérat EM, Cowan NC, Gakis G (2021). European association of urology guidelines on muscle-invasive and metastatic bladder cancer: summary of the 2020 guidelines. Eur Urol.

[12] Rice KR, Koch MO, Kao CS, Pedrosa JA, Kaimakliotis HZ, Masterson TA (2015). Lymph node metastases in patients with urothelial carcinoma variants: influence of the specific variant on nodal histology. Urol Oncol.

[13] Amin MB, Greene FL, Edge SB, Compton CC, Gershenwald JE, Brookland RK (2017). The eighth edition AJCC cancer staging manual: continuing to build a bridge from a population-based to a more "personalized" approach to cancer staging. CA Cancer J Clin.

[14] World Medical Association Declaration of Helsinki (2013). Ethical principles for medical research involving human subjects. JAMA.

[15] Black AJ, Black PC (2020). Variant histology in bladder cancer: diagnostic and clinical implications. Transl Cancer Res.

[16] Ganeshalingam S, Koh DM (2009). Nodal staging. Cancer Imaging.

[17] Herr HW (2003). Superiority of ratio based lymph node staging for bladder cancer. J Urol.

[18] Kim HS, Moon KC, Jeong CW, Kwak C, Kim HH, Ku JH (2015). Histological variant as the significant predictor of survival in patients with lymph node positive urothelial carcinoma of the bladder. Sci Rep.

[19] Vetterlein MW, Wankowicz SAM, Seisen T, Lander R, Löppenberg B, Chun FK (2017). Neoadjuvant chemotherapy prior to radical cystectomy for muscle-invasive bladder cancer with variant histology. Cancer.

[20] Processali T, Diminutto A, Cerruto MA, Antonelli A (2020). The impact of histological variants on bladder cancer outcomes. AME Med J.

[21] Al-Alao O, Mueller-Leonhard C, Kim SP, Amin A, Tucci C, Kott O (2020). Clinically node-positive (cN+) urothelial carcinoma of the bladder treated with chemotherapy and radical cystectomy: clinical outcomes and development of a postoperative risk stratification model. Urol Oncol Semin Orig Investig.

[22] Horn T, Zahel T, Adt N, Schmid SC, Heck MM, Thalgott MK (2016). Evaluation of computed tomography for lymph node staging in bladder cancer prior to radical cystectomy. Urol Int.

[23] Shankar PR, Barkmeier D, Hadjiiski L, Cohan RH (2018). A pictorial review of bladder cancer nodal metastases. Transl Androl Urol.

[24] Schmid SC, Zahel T, Haller B, Horn T, Metzger I, Holzapfel K (2016). Prognostic value of computed tomography before radical cystectomy in patients with invasive bladder cancer: imaging predicts survival. World J Urol.

[25] Baumeister P, Zamboni S, Mattei A, Antonelli A, Simeone C, Mordasini L (2019). Histological variants in non-muscle invasive bladder cancer. Transl Androl Urol.

[26] Song J, Yin Y, Wang H, Chang Z, Liu Z, Cui L (2020). A review of original articles published in the emerging field of radiomics. Eur J Radiol.

