# Supplementary material for: Accuracy and prognostic value of radiological lymph node features in variant histologies of bladder cancer
Source: World J Urol. 2022 Apr 23;40(7):1707–14. doi: 10.1007/s00345-022-04010-6 (PMC9236971; doi:10.1007/s00345-022-04010-6)
Supplement: Supplementary file 1 — Supplementary file1 (DOCX 1331 kb) [file 345_2022_4010_MOESM1_ESM.docx]

**Supplementary**

**
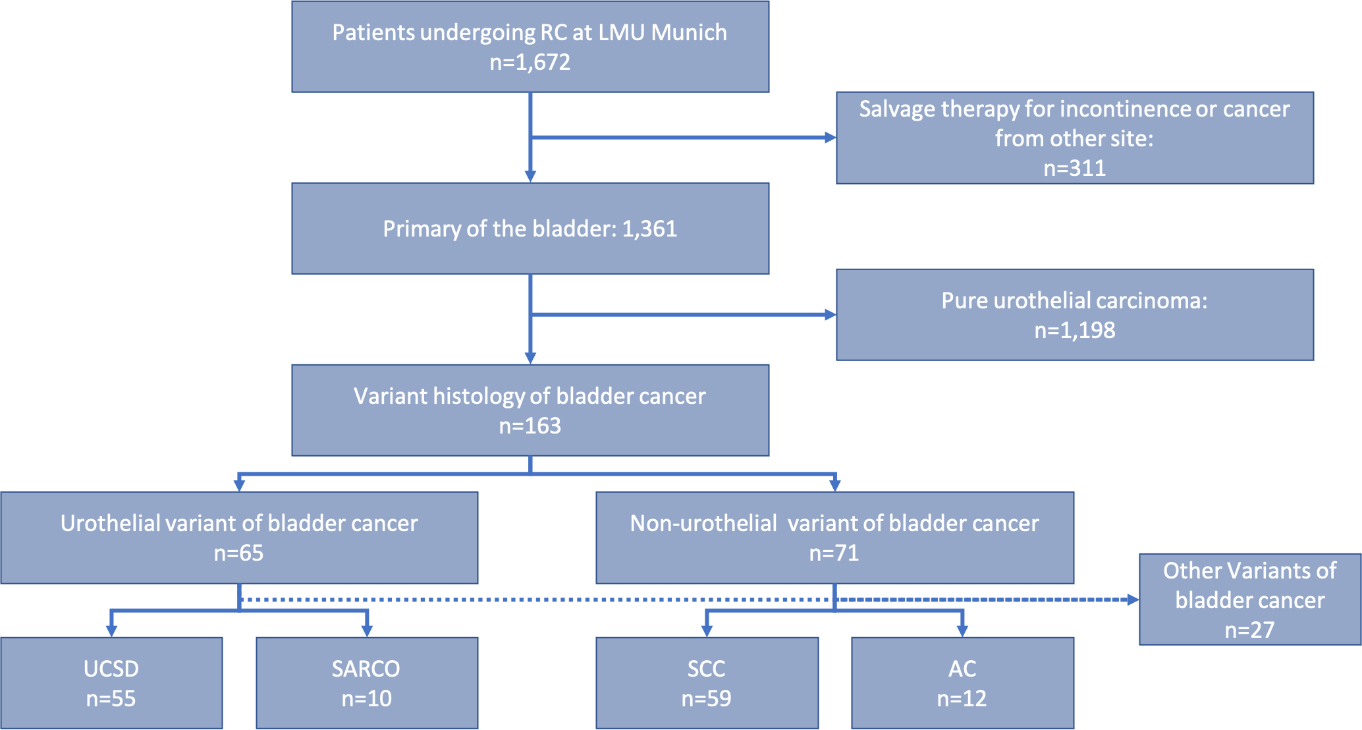
Supplementary figure 1: Patient identification**

Abbr.: UCSD: urothelial carcinoma with squamous differentiation, SARCO: sarcomatoid urothelial carcinoma SCC: squamous cell carcinoma, AC: Adenocarcinoma, RC: radical cystectomy, n: number of patients.

**Supplementary figure 2: CSS of variant histologies of bladder cancer**


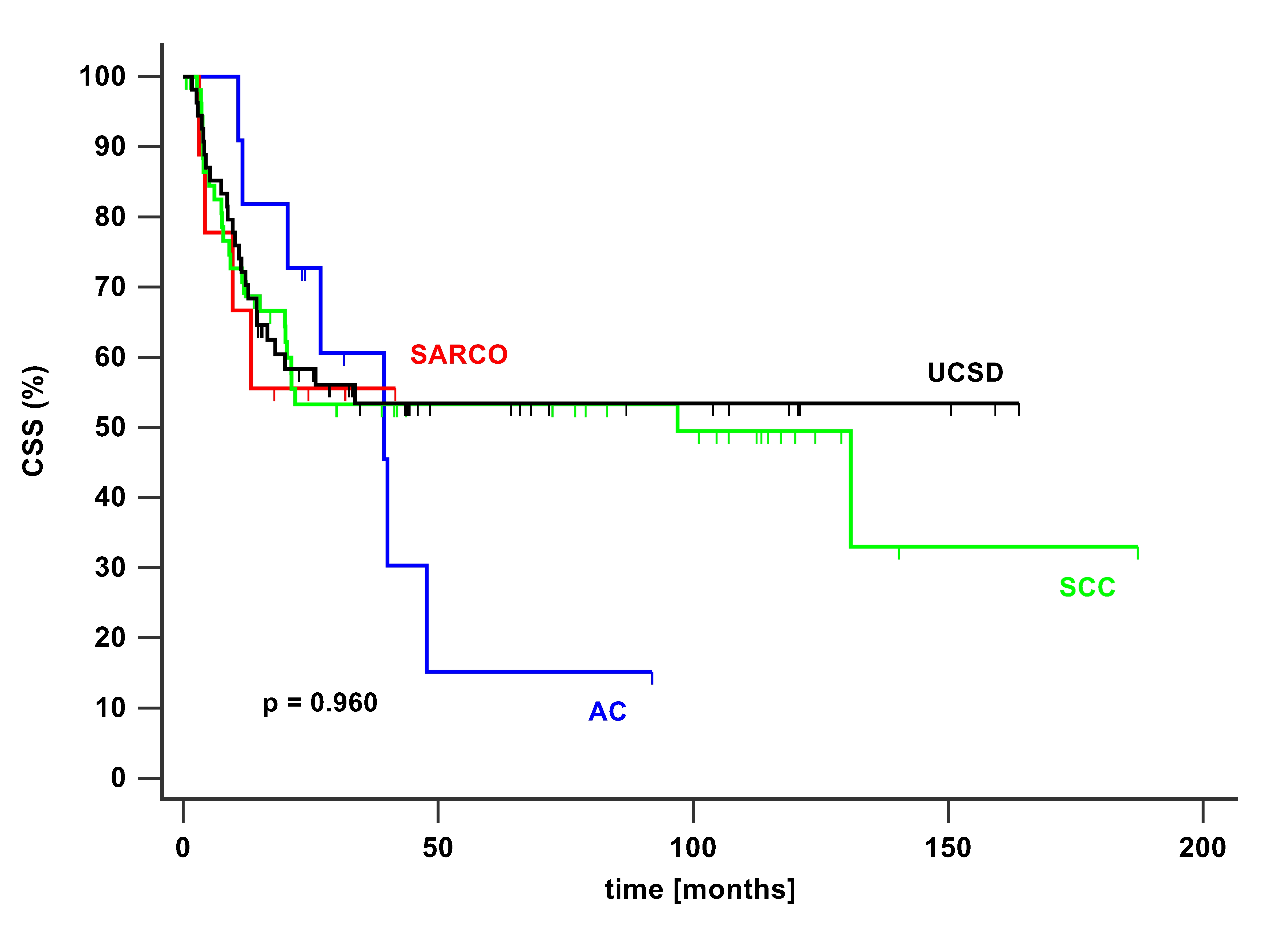


Cancer specific survival (CSS) for depicted variant histologies of cancer calculated by Kaplan-Meier method. Abbr. UCSD urothelial carcinoma with squamous differentiation, SARCO: sarcomatoid urothelial carcinoma, SCC: squamous cell carcinoma, AC: Adenocarcinoma.


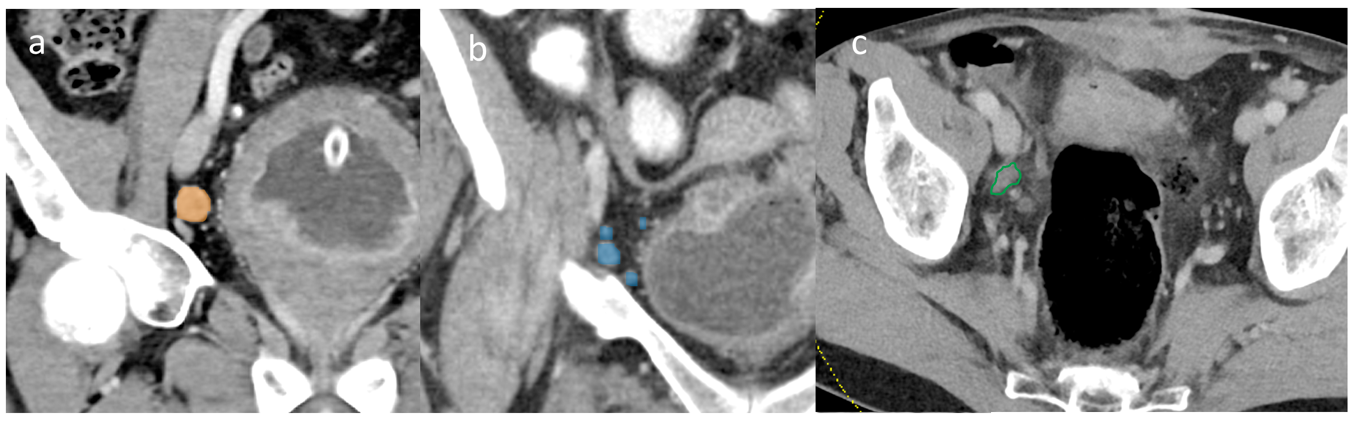
**Supplementary figure 3: Representative CT morphological characteristics of Lymph nodes**

Representative Lymph node characteristics in CT scan. Enlarged lymph node are shown in coronal plane and marked in orange (a). Increased number of loco-regional lymph nodes in coronal plane are highlighted in blue color (b). A representative lymph node with irregular configuration and loss of fatty hilum in transversal plane is outlined in green (c).

**Supplementary figure 4: Accuracy per size of lymph nodes**

Receiver operating characteristics (ROC) analysis was performed for sensitivity and specificity as per size of lymph nodes.

Abbr.: LN: lymph node, AUC: area under the curve, p: p-value.

**Supplementary table 1: Multivariate analysis of predictors of survival**

|  | **HR** | **95% CI of HR** | **p value** |
| --- | --- | --- | --- |
| **Increased number** | 2.190 | 0.705 to 6.803 | 0.175 |
| **Fatty hilum** | 0.313 | 0.104 to 0.945 | 0.039 |
| **Age** | 1.009 | 0.970 to 1.050 | 0.655 |
| **Gender_(male)** | 0.779 | 0.314 to 1.932 | 0.590 |
| **pT3/4** | 7.608 | 0.887 to 65.285 | 0.064 |
| **pN+** | 2.866 | 1.140 to 7.207 | 0.025 |
